# 

**Published:** 1874-03

**Authors:** 


### Contents of March Number.

### ORIGINAL DEPARTMENT.

ACT. I.—On The Diffusion of Typhoid Fever by means of drinking water. By Austin Flint, M. D. ........ ........................ 281
ART. II.—Thoughts on Medical Progress. By T. D. Washburn, M. D... 294
ART. III.—Medical Society of the County of Albany, Semi-Monthly Meeting. Reported by F. C. Curtis, M. D., ...................... 300

### MISCELLANEOUS.

The Regulations of Insane Asylums, ........................... ... 305
A Medical License for Ladies, .................................... 305
Address before the Graduating Class of the Buffalo Medical College, February 24th, 1874. By Joseph Warren, Esq., ...................... 306

### EDITORIAL DEPARTMENT.

Editorial Change, ........................................... 314
Dr. Bela H. Colgrove, ....................... ..................... 315
A Doctor needing Reconstruction ................................. 317
Hairy Man, ............ ..................... ................. 318

Books Reviewed:

A Practical Treatise on Diseases of Children. By Drs. Meigs & Pepper, .......................................... 318
An Introduction to the study of Clinical Medicine, .......... 319
Clinical Researches in Electro-Surgery. By Drs. Beard & Rockwell, .......... ............................ .. 219

Books and Pamphlets Received ..... .. ........................... 320

